# Elucidating the Relationship between Neutrophil–Lymphocyte Ratio and Plaque Composition in Patients with Drug-Eluting Stent Restenosis by Virtual Histology-Intravascular Ultrasound

**DOI:** 10.3390/jcdd11070211

**Published:** 2024-07-04

**Authors:** Ming Yu, Yuxing Wang, Song Yang, Jiajie Mei, Zhenzhu Liu, Lijiao Zhang, Wenli Xie, Zhaohong Geng, Baole Liu, Hongyan Wang, Peng Qu, Nan Niu

**Affiliations:** 1 The First Department of Cardiology, The Second Affiliated Hospital of Dalian Medical University, Dalian 116023, China; ym_0630@aliyun.com (M.Y.);; 2 Department of Medicine, Dalian University of Technology, Dalian 116081, China

**Keywords:** in-stent restenosis, neutrophil–lymphocyte ratio, virtual histology-intravascular ultrasound, plaque composition

## Abstract

(1) Background: In-stent Restenosis (ISR) is a major factor influencing the prognosis and revascularization of target lesions. The plaque composition is unclear; therefore, it is critical to investigate ISR composition to identify clinical intervention markers. (2) Methods: This study was conducted on 36 patients with drug-eluting stent restenosis. The patients were classified into a Low Neutrophil–Lymphocyte Ratio (L-NLR) and High Neutrophil–Lymphocyte Ratio (H-NLR) according to the median NLR level of 36 patients. Discrepancies in the current information such as baseline data, biochemical examination, cardiac ultrasound data, etc., were examined to identify the underlying risk factors, and a multifactorial linear regression analysis of plaque properties was conducted. (3) Results: NLR = 2.64 was utilized to classify 18 patients into the L-NLR group and 18 patients into the H-NLR group. There were statistically significant differences in age, a pre-percutaneous coronary intervention (PCI) SYNTAX II score, a C-reactive protein (CRP), interleukin (IL)-6, plaque loading, a fibro-lipid tissue area, calcified nubs, and virtual histology-thin fibrous cap atherosclerotic (VH-TCFA). The significant impacts of variations in age, neutrophil–lymphocyte ratio (NLR) levels, and IL-6 levels on the plaque stress and percentage of the fibro-lipid tissue in virtual histology-intravascular ultrasound (VH-IVUS) were identified through multifactorial linear regression. (4) Conclusions: The high NLR group demonstrated increased myocardial injury severity, consistent with higher SYNTAX II scores, a higher plaque burden, and higher proportions of vulnerable components. NLR proved to be a risk factor for both the plaque load and the proportion of the fibro-lipid tissue in ISR.

## 1. Introduction

The incidence of coronary atherosclerotic heart disease (CHD) has steadily increased in recent years due to changes in dietary habits and lifestyle practices. Worldwide, CHD has been associated with a high mortality rate. Advances in drug-eluting stents (DES) have substantially improved the efficacy and outcomes of percutaneous coronary interventions (PCIs). However, the repeated target lesion revascularization (TLR) rate has been reported to be 1–2% per year [1,2]. It has been demonstrated that PCIs for In-stent Restenosis (ISR) is associated with a higher probability of serious adverse cardiac events, including myocardial infarction, stent thrombosis, and all-cause mortality, in comparison to PCIs for de novo lesions [2,3,4]. Since millions of DES are implanted annually worldwide, ISR remains an inevitable clinical concern. After a PCI, a portion of significant adverse cardiovascular events can be avoided by decreasing ISR.

ISR is a healing process wherein the body over-reacts to an external injury or stimulus. It is different from the chronic progression of coronary atherosclerosis. Restenosis occurs in response to an exaggerated vascular injury. Vascular elastic regression and thrombosis usually occur within minutes to hours after PCIs. In contrast, neointimal hyperplasia, inflammatory responses, vascular remodeling, and neoarterial endothelial atherosclerosis are manifested in weeks to months after PCIs. Drug-coated stents exert slow and sustained anti-inflammatory and anti-proliferative effects [2,5]. While ISR occurrence is significantly delayed with DES compared to that observed with bare-metal stents, it is still not eliminated [6]. The recurrence rate of in-stent restenosis remains high even after effective treatment, and the long-term prognosis varies with the choice of the treatment modality [7]. Studies have shown that DES delays endothelial repair, stimulates a vascular endothelial inflammatory response, and promotes ISR development [8,9]. In summary, the mechanism underlying the occurrence of ISR after DES implantation is complex.

Leukocytes and their subpopulations have gained scientific attention as indicators of inflammation [10]. A novel composite inflammatory marker associated with leukocytes is the neutrophil–lymphocyte ratio (NLR). Any change in the NLR may indicate an inflammatory response linked to cardiovascular diseases, and an increased NLR level suggests a hyperinflammatory response. Previous reports have confirmed a close relationship between the NLR and ISR after PCI [11,12,13], and there are only a few reports on the nature of the plaque in drug-coated stents. To provide new evidence for the mechanism underlying ISR and to suggest clinical intervention cues, we use VH-IVUS to examine the relationship between various NLR levels and the nature of ISR plaque.

## 2. Materials and Methods

### 2.1. Study Subjects

This study is a double-blind cross-sectional prospective study. We thoroughly analyzed the clinical data of consecutive patients with chronic or acute coronary syndrome who visited the Department of Cardiology I, Second Affiliated Hospital of Dalian Medical University, from December 2022 to October 2023. The subjects were categorized into two groups based on median of NLR levels [14,15]. This analysis aimed to improve the accuracy of coronary angiography in understanding the ISR process. This study received approval from the Ethics Committee of the Second Affiliated Hospital of Dalian Medical University. All participants provided written consent after being informed about the study. All experiments were conducted following proper guidelines and regulations.

#### 2.1.1. Inclusion Criteria

(1) Age, 18–85 years; (2) first PCI significantly improved coronary stenosis [residual stenosis < 30%, thrombolysis in myocardial infarction (TIMI) grade III], and all of them were drug-eluting stents; (3) clear ISR in the follow-up of coronary angiography (criterion: post-PCI follow-up shows >50% stenosis of the diameter of the vessel in the stent or within the edge of the stent within 5 mm); and (4) willingness to participate in the study and co-operate with the investigator during the operation.

#### 2.1.2. Exclusion Criteria

(1) Pregnancy, severe liver, and kidney insufficiency, or acute and chronic infections; (2) malignant neoplastic diseases, Alzheimer’s disease, inflammatory bowel disease, and other autoimmune diseases; (3) patients with previous coronary artery bypass grafting; (4) patients who have experienced severe trauma, major surgery, or cardiopulmonary resuscitation in the previous 6 months; (5) patients on hormone or other immune inhibitor therapies for a long time; (6) those with other vascular progressions at the time of follow-up; and (7) those with lesions that could not be passed by the ultrasound probe owing to severe twisting or stenosis.

### 2.2. Clinical Baseline Data Collection

Data were collected from patients who met the inclusion criteria and comprised gender, age, past medical history such as hypertension and diabetes mellitus, admission blood pressure, smoking history, and drug application.

### 2.3. Biochemical Tests and Blood Cell Counts

On the 2nd day of hospitalization, early-morning fasting elbow venous blood samples were collected from all patients. These samples were used to test for myocardial injury markers such as creatine kinase MB (CK-MB), troponin I (cTnI), B-type natriuretic peptide (BNP), and N-terminal pro-B-type natriuretic peptide NT-proBNP(NT-proBNP). Additionally, routine blood tests were conducted using the MINDRAYBC6800 (Mahwah, NJ, USA) machine to measure white blood cell (WBC) count, red blood cell (RBC) count, hemoglobin (Hb) levels, and platelet (PLT) count. Liver biochemistry tests were performed using the SIEMENS (Plano, TX, USA) ADVIA2400 equipment to measure alanine transaminase (ALT), aspartate transaminase (AST), albumin, gamma-glutamyl transferase, lactate dehydrogenase, and blood lipids such as total cholesterol (TC), triglyceride (TG), high-density lipoprotein cholesterol (HDL-C), low-density lipoprotein (LDL), ApoA, and ApoB. Other parameters measured include fasting blood glucose (FBG), glycosylated hemoglobinA1c (HbA1c), urea, creatinine, uric acid, cystatin C, thyroid-stimulating hormone (TSH), and thyroxine T4 (fT4). Pro-inflammatory cytokines, including interleukin(IL)-1β, IL-6, and tumor necrosis factor (TNF)-α, were measured using the Siemens Test Kit.

### 2.4. Cardiac Echocardiography

A specialized cardiac sonographer conducted cardiac echocardiography using the American GE (Addison, TX, USA) Logig7 color Doppler ultrasound. The purpose was to measure various parameters, including atrial and ventricular diameters, interventricular septal thickness (IVST), peak mitral inflow velocities in early diastole, the average value of the mitral annular velocities in early diastole (Em), left ventricular ejection fraction (LVEF), left ventricular posterior wall thickness, left ventricular weight index, mitral orifice flow velocity E, E/A ratio, mitral annular septal side e′, mitral annular side wall side e′, and mean E/e′.

### 2.5. Coronary Angiography and Intravascular Ultrasound (IVUS)

All patients were subjected to coronary angiography by experienced interventionist physicians in the Department of Cardiovascular Medicine in this study according to the Judkins technique. Before using IVUS from Volcano, a dosage of 0.2 mg of nitroglycerin was administered into the coronary artery through a guide catheter. Then, an ultrasound catheter was sent through the stenotic lesion and over the distal end of the stent. Images were captured until it was over the proximal edge of the stent while being retracted. The virtual histology (VH) program was used to determine the plaque’s composition [16]. The fibrous tissue was indicated by the color green, the fibro-lipid tissue by yellow–green, the calcified tissue by white, and the necrotic tissue components by red. Virtual histology- thin fibrous cap atherosclerotic (VH-TCFA) plaques were identified where the necrotic tissue components comprised more than 10% of the vessel segment with over 40% plaque buildup in the luminal cross-section. In these plaques, the necrotic nuclei were directly in contact with the lumen, and there was no fibrous cap on the surface in three consecutive frames of VH-IVUS images [17,18].

### 2.6. Statistical Analysis

SPSS 26.0 statistical software was used to analyze the results. Measurement data that followed a normal distribution were expressed as mean ± standard deviation ($\bar{x}$ ± SD), while those that did not obey the normal distribution were expressed as median (Quartile 1, Quartile 3) [M (Q1, Q3)]. Comparison between two groups was performed using a *t*-test or a non-parametric rank sum test. A chi-square test was utilized to compare groups, and count data were reported as cases (%). A multifactor linear regression analysis was also carried out. All tests were two-sided; differences were considered statistically significant at *p* < 0.05.

## 3. Results

### 3.1. Patient Status

A total of 36 ISR patients were enrolled. Their mean (standard deviation) age was 68 (±8) years, and their body mass index (BMI) was 25.25 (±2.56) Kg/m^2^. Of them, 25 were male (69.4%). Based on their median NLR level (NLR = 2.64), 18/36 patients were categorized into the L-NLR group and 18 into the H-NLR group. 

### 3.2. Comparison of the General Clinical Characteristics of the Patients from the Two Groups (Table 1)

There were no significant differences in gender, height, weight, BMI, smoking status, alcohol consumption, hypertension, admission systolic blood pressure (SBP), admission diastolic blood pressure (DBP), diabetes mellitus, and combined myocardial infarction between the two groups. However, the two groups showed statistically significant differences in their age (*p* < 0.05).

**Table 1:** Comparison of the overall clinical features between the two groups.

Characteristics	L-NLR (*n* = 18)	H-NLR (*n* = 18)	*p*
Male (%)	13 (72.2)	12 (66.7)	0.717
Age (years)	65.61 ± 9.08	71.22 ± 6.83	0.044 *
Height (cm)	168.44 ± 7.79	168.17 ± 6.86	0.910
Weight (kg)	73.17 ± 10.97	70.28 ± 9.00	0.394
BMI (kg/m^2^)	25.68 ± 2.69	24.81 ± 2.40	0.311
Smoking (%)	8 (44.4)	9 (50.0)	0.738
Alcohol (%)	3 (16.7)	0 (0.00)	0.070
Hypertension (%)	16 (88.9)	13 (77.2)	0.206
SBP (mmHg)	128.89 ± 17.02	134.44 ± 20.19	0.378
DBP (mmHg)	81.06 ± 10.84	79.11 ± 15.29	0.663
Diabetes mellitus (%)	10 (55.6)	13 (72.2)	0.298
Combined myocardial infarction (%)	13 (72.2)	15 (83.3)	0.423

* Statistically significant difference (*p* < 0.05).

### 3.3. Comparison of Medications between the Two Groups (Table 2)

Aspirin/indobufen, clopidogrel/Ticagrelor, and statins were prescribed to patients from both groups. There were no differences in the prescriptions of drugs such as ezetimibe, Proprotein convertase subtilisin/kexin type 9 (PCSK9) inhibitors, beta-blockers, angiotensin-converting enzyme inhibitor (ACEI)/angiotensin receptor blocker (ARB)/angiotensin receptor and neprilysin inhibitor (ARNI), calcium antagonists, nicorandil, sodium-dependent glucose transporters 2 (sGLT-2) inhibitors, and liraglutide between the two groups.

**Table 2:** Comparison of the drugs administered to both groups.

Characteristics	L-NLR (*n* = 18)	H-NLR (*n* = 18)	*p*
Aspirin/indobufen (%)	18 (100.0)	18 (100.0)	-
Clopidogrel/Ticagrelor (%)	18 (100.0)	18 (100.0)	-
Statins (%)	18 (100.0)	18 (100.0)	-
Ezetimibe (%)	6 (33.3)	5 (27.8)	0.717
PCSK9 inhibitors (%)	6 (33.3)	5 (27.8)	0.717
Beta-blockers (%)	10 (55.6)	12 (66.7)	0.494
ACEI/ARB/ARNI (%)	9 (50.0)	10 (55.6)	0.738
Calcium antagonists (%)	4 (22.2)	3 (16.7)	0.674
Nicorandil (%)	10 (55.6)	12 (66.7)	0.494
sGLT-2 inhibitors (%)	9 (50.0)	6 (33.3)	0.310
Liraglutide (%)	6 (33.3)	6 (33.3)	0.310

### 3.4. Comparison of Biochemical Indices between the Two Groups (Table 3)

There were no significant differences between the two groups in terms of RBC, Hb, PLT, FBG, HbA1c, TC, TG, HDL-C, LDL-C, ApoA, ApoB, urea, creatinine, uric acid, cystatin C, ALT, AST, albumin, γ-glutamyltransferase (γ-GGT), TSH, and fT4. However, significant differences were observed in the levels of CK-MB, cTnI, BNP, NT-proBNP, WBC, alkaline phosphatase, and lactate dehydrogenase (*p* < 0.05).

**Table 3:** Comparison of biochemical indices and blood cell counts between the two groups.

Characteristics	L-NLR (*n* = 18)	H-NLR (*n* = 18)	*p*
CK-MB (ug/mL)	1.25 (1.00, 1.48)	1.30 (0.83, 60.60)	0.009 *
cTnI (ug/mL)	0.008 (0.004, 0.09)	0.027 (0.002, 35.89)	0.002 *
BNP (pg/mL)	47.85 (15.53, 242.2)	129.95 (28.50, 384.9)	0.035 *
NT-proBNP (pg/mL)	108.20 (104.48, 745.08)	162.10 (107.78, 1352.18)	0.011 *
WBC (10^9^/L)	5.87 ± 1.88	7.43 ± 2.26	0.031 *
Neutrophil (10^9^/L)	3.59 ± 0.92	5.66 ± 1.97	<0.001
Lymphocyte (10^9^/L)	2.06 ± 0.78	1.25 ± 0.44	<0.001
Neutrophil–lymphocyte ratio	1.85 ± 0.50	4.93 ± 2.04	<0.001
RBC (10^12^/L,)	4.55 ± 0.48	4.28 ± 0.59	0.136
Hb (g/L)	140.00 ± 15.30	129.72 ± 20.06	0.093
PLT (10^9^/L)	232.17 ± 66.23	201.94 ± 64.31	0.174
FBG (mmol/L)	6.43 ± 1.56	7.19 ± 2.72	0.253
HbA1c (%,)	7.13 ± 1.55	7.40 ± 1.28	0.572
TC (mmol/L)	3.77 ± 1.72	3.79 ± 1.15	0.971
TG (mmol/L)	1.97 ± 1.67	1.44 ± 0.76	0.231
HDL-C (mmol/L)	0.93 ± 0.23	0.95 ± 0.20	0.805
LDL-C (mmol/L)	2.02 ± 1.21	2.27 ± 0.99	0.504
ApoA (mmol/L)	1.23 ± 0.19	1.17 ± 0.17	0.313
ApoB (mmol/L)	0.72 ± 0.34	0.74 ± 0.23	0.794
Urea nitrogen (mmol/L)	6.17 ± 1.48	7.63 ± 5.59	0.294
Creatinine (μmol/L)	65.42 (61.58, 83.10)	81.32 (62.44, 106.18)	0.206
Uric acid (μmol/L)	368.52 ± 89.85	337.41 ± 96.94	0.325
Cystatin C(pg/L)	1.20 (1.03, 1.27)	1.23 (0.89, 1.75)	0.498
ALT (U/L)	21.97 ± 7.92	26.16 ± 14.75	0.243
AST (U/L)	19.06 (17.18, 21.50)	17.88 (12.92, 79.66)	0.076
Albumin (g/L)	39.72 ± 4.32	35.29 ± 9.29	0.075
γ-GGT (U/L)	23.24 ± 8.14	19.21 ± 8.07	0.146
Alkaline phosphatase (U/L)	90.29 ± 30.48	67.47 ± 16.41	0.008 *
Lactate dehydrogenase (U/L)	179.28 ± 30.88	316.92 ± 172.83	0.002 *
TSH (mIU/L)	1.68 ± 1.44	1.91 ± 1.69	0.657
fT4 (pmol/l)	14.43 ± 2.39	13.27 ± 2.06	0.126

* Statistically significant difference between the two groups.

### 3.5. Comparison of Cardiac Echocardiography between the Two Groups (Table 4)

No significant differences were evident in the left atrium diameter, left ventricular-end diastolic internal diameter, septal thickness, right atrial internal diameter, right ventricular internal diameter, left ventricular posterior wall thickness, left ventricular weight index, ejection fraction, mitral orifice flow velocity E, E/A, mitral annular septal side e′, mitral annular side wall side e′, and mean E/e′ between the two groups. However, the two groups had a significantly different mitral orifice blood flow velocity A (*p* < 0.05).

**Table 4:** Comparison of cardiac echocardiography between the two groups.

Characteristics	L-NLR (*n* = 18)	H-NLR (*n* = 18)	*p*
Left atrium diameter (mm)	38.92 ± 4.29	38.84 ± 4.37	0.534
Left ventricular-end diastolic internal diameter (mm)	46.75 ± 5.08	45.71 ± 3.91	0.500
Left ventricular-end systolic internal diameter (mm)	29.56 ± 7.39	28.82 ± 4.51	0.720
Septal thickness (mm)	10.69 ± 0.99	10.89 ± 1.30	0.614
Right atrial internal diameter (mm)	35.24 ± 3.49	34.84 ± 1.75	0.745
Right ventricular internal diameter (mm)	10.46 ± 0.88	11.48 ± 3.46	0.601
Left ventricular posterior wall thickness (mm)	10.46 ± 0.88	11.48 ± 3.46	0.250
Left ventricular weight index (g/m^2^)	97.67 ± 18.22	103.97 ± 23.38	0.432
Ejection fraction (%)	55.59 ± 7.82	55.06 ± 6.87	0.832
E (cm/s)	65.76 ± 18.13	69.47 ± 13.09	0.499
A (cm/s)	85.65 ± 14.60	97.35 ± 13.89	0.023 *
E/A	0.78 ± 0.22	0.72 ± 0.16	0.420
Septal side e′ (cm/s)	6.11 ± 1.23	5.51 ± 1.37	0.256
Sidewall side e′ (cm/s)	8.43 ± 1.64	8.43 ± 1.84	0.896
mean E/e′	9.21 ± 2.89	10.13 ± 2.20	0.373

* Statistically significant difference between the two groups.

### 3.6. Comparison of Coronary Angiography Results between the Two Groups (Figure 1 and Table 5)

The two groups showed statistically significant differences in the SYNTAX II score and Mehran in-stent restenosis classification (*p* < 0.05). The Mehran classification was predominantly type I and II for the L-NLR group and type I and II for the H-NLR group. No significant differences were observed in the number of branches with previous lesions, type of stent drug coating, pre-procedural Gensini score, SYNTAX score before the first PCI, number of branches with the current lesion, number of stents, stent diameter, stent length, and interval duration.

**Figure 1:**
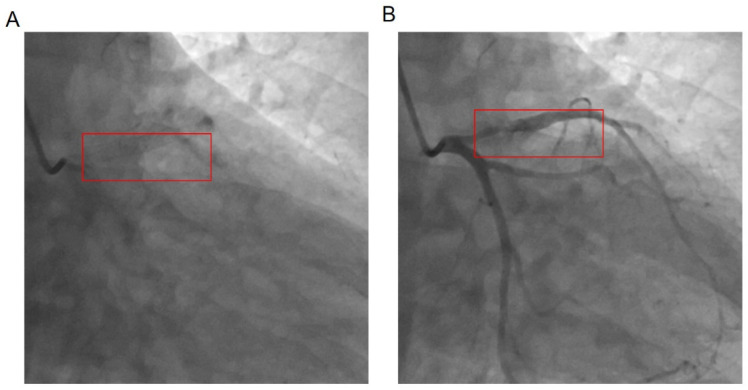
Coronary angiography stent and stenosis location. (**A**) Stent position, (**B**) in-stent restenosis position.

**Table 5:** Comparison of coronary angiography results between the two groups.

Characteristics	L-NLR (*n* = 18)	H-NLR (*n* = 18)	*p*
Previous lesions (%)			
three-branch lesions	2 (11.1)	3 (16.7)	0.630
two-branch lesions	9 (50.0)	9 (50.0)	1.000
single-branch lesion	7 (38.9)	6 (33.3)	0.729
Drug Coating (%)			
Paclitaxel	2 (11.1)	2 (11.1)	1.000
Rapamycin	5 (27.8)	8 (44.4)	0.298
Sirolimus/everolimus/ zotarimus	13 (72.2)	8 (44.4)	0.091
Pre-PCI Gensini score	47.83 ± 25.73	57.75 ± 29.23	0.288
Pre-PCI SYNTAX score	11.89 ± 9.02	12.58 ± 5.79	0.785
Pre-PCI SYNTAX II score	21.92 ± 5.66	27.99 ± 10.42	0.037 *
Current lesion (%)			
Trichobranchial lesions	0 (0.00)	1 (5.6)	0.310
Branchial lesions	1 (5.6)	0 (0.00)	0.310
Single lesion	17 (94.4)	17 (94.4)	1.000
LAD	9 (50.0)	11 (61.1))	
LCX	2 (11.1)	2 (11.1)	
RCA	6 (33.3)	4 (22.2)	
Mehan classification			0.027 *
class I (%)	6 (33.3)	1 (5.6)	0.035 *
class II (%)	10 (55.6)	9 (50.0)	0.738
class III (%)	2 (11.1)	8 (44.4)	0.026 *
class IV (%)	0 (0.00)	0 (0.00)	-
Number of stents	2.94 ± 1.39	3.27 ± 1.67	0.552
Stent Diameter (cm)	3.07 ± 0.20	3.11 ± 0.33	0.690
Stent length (cm)	19.39 ± 5.21	21.50 ± 5.89	0.331
Interval duration (years)	6.59 ± 3.99	7.36 ± 5.34	0.630

* Statistically significant difference between the two groups. LAD, left anterior descending coronary artery. LCX, left circumflex coronary artery. RCA, right coronary artery.

### 3.7. Comparison of Pro-Inflammatory Indicators between the Two Groups (Table 6)

The levels of C-reactive protein (CRP), IL-1β, IL-6, and TNF-α were statistically significantly different (*p* < 0.05) between the two groups. However, no significant differences were observed in the interleukin 2 receptor (IL-2R) and IL-8 levels. 

**Table 6:** A comparison of pro-inflammatory indicators between the two groups.

Characteristics	L-NLR (*n* = 18)	H-NLR (*n* = 18)	*p*
CRP (pg/mL)	1.10 (1.04, 2.24)	4.90 (2.94, 21.25)	0.016 *
IL-1β (pg/mL)	5.00 (5.00, 6.30)	9.24 (5.79, 12.05)	0.017 *
IL-2R (pg/mL)	365.00 (333.25, 472.50)	425 (361.00, 763.00)	0.421
IL-6 (pg/mL)	6.70 (2.90, 16.28)	15.70 (8.76, 26.65)	0.044 *
IL-8 (pg/mL)	239.00 (67.10, 706.25)	368.00 (172.25, 715.50)	0.118
TNF-α (pg/mL)	12.35 (11.48, 74.05)	66.30 (29.45, 104.40)	0.012 *

* Statistically significant difference.

### 3.8. Comparison of Intravascular Ultrasound Grayscale Data and VH-IVUS Plaque Composition between the Two Groups (Figure 2 and Table 7)

The minimum lumen area, plaque load, minimal lumen external elastic membrane area, fibrous tissue proportion, fibro-lipid tissue area, fibro-lipid tissue proportion, calcified nodule proportion, and VH-TCFA plaque proportion were statistically significantly different between the two groups (*p* < 0.05). The minimal lumen diameter, the minimal lumen in-stent area, the eccentricity index, the minimal luminal external elastic membrane area, the proximal and distal reference end external elastic membrane area, the remodeling index, the proximal and distal reference end in-stent area, the reference lumen area, the fibrous tissue area, the necrotic core area, the necrotic core proportion, the calcified tissue area, and the calcified tissue proportion, however, did not show any significant differences. 

**Figure 2:**
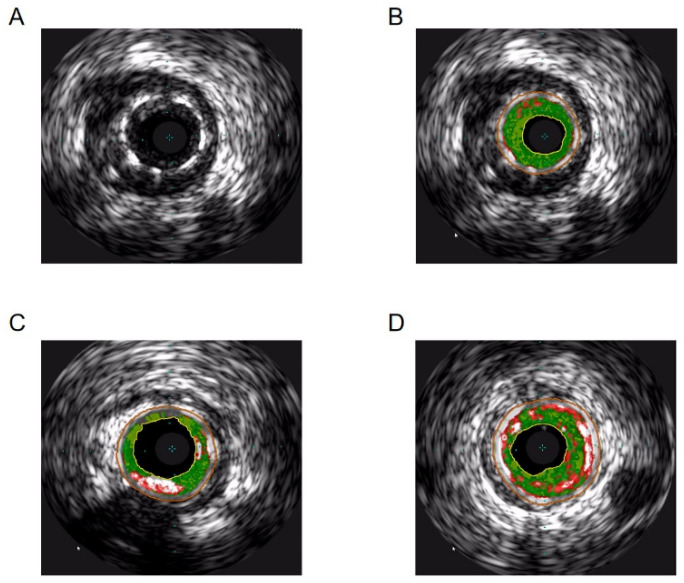
A concise overview of the standard VH-IVUS observations. (**A**) Grayscale image of in-stent restenosis, (**B**) composition of plaques under VH-IVUS, (**C**) calcified nodules, and (**D**) VH-TCFA. The fibrous tissue was indicated by the color green, the fibro-lipid tissue by yellow–green, the calcified tissue by white, and the necrotic tissue components by red.

**Table 7:** Comparison of intravascular ultrasound grayscale data and VH plaque composition between the two groups.

Characteristics	L-NLR (*n* = 18)	H-NLR (*n* = 18)	*p*
Minimum lumen diameter (mm)	1.65 ± 0.23	1.60 ± 0.23	0.563
Minimum lumen in-stent area (mm^2^)	9.38 ± 2.89	7.61 ± 1.81	0.087
Minimum lumen area (mm^2^)	2.99 ± 1.55	1.96 ± 0.50	0.044 *
Plaque load (%)	69.19 ± 5.422	76.79 ± 4.57	0.001 *
Eccentricity index	4.26 ± 3.16	5.69 ± 3.21	0.271
Minimum lumen external elastic membrane area (mm^2^)	20.97 ± 4.28	17.32 ± 4.45	0.045 *
Proximal reference end external elastic membrane area (mm^2^)	20.42 ± 5.14	18.22 ± 2.00	0.213
Distal reference end external elastic membrane area (mm^2^)	13.80 ± 5.06	13.34 ± 3.58	0.806
Remodeling index	1.38 ± 0.67	1.22 ± 0.38	0.413
Proximal reference end stent internal area (mm^2^)	10.37 ± 2.78	11.66 ± 3.69	0.328
Lumen area (mm^2^)	6.95 ± 3.42	8.47 ± 4.74	0.359
Distal reference end stent internal area (mm^2^)	7.91 ± 1.80	9.55 ± 4.62	0.223
Lumen area (mm^2^)	5.00 ± 1.37	7.58 ± 5.00	0.070
Fibrous tissue area (mm^2^)	3.01 ± 1.66	1.90 ± 1.02	0.068
Fibrous tissue proportion (%)	56.62 ± 12.39	42.20 ± 18.05	0.031 *
Fibro-lipid tissue area (mm^2^)	1.16 ± 0.65	2.06 ± 1.17	0.027 *
Fibro-lipid tissue proportion (%)	22.98 ± 13.14	41.66 ± 19.06	0.010 *
Necrotic core area (mm^2^)	0.77 ± 0.61	0.89 ± 0.78	0.679
Necrotic core proportion (%)	14.60 ± 8,96	14.94 ± 9.57	0.930
Calcified tissue area (mm^2^)	0.30 ± 0.23	0.14 ± 0.11	0.050
Calcified tissue proportion (%)	5.66 ± 5.44	3.51 ± 2.91	0.253
Calcified nodules (%)	3 (16.7)	7 (38.9)	0.040 *
VH-TCFA (%)	2 (11.1)	7 (38.9)	0.049 *

* Statistically significant difference between the two groups.

### 3.9. Multifactorial Regression Analysis of VH-IVUS Plaque Components between the Two Groups (Table 8, Table 9 and Table 10)

We constructed a multifactorial linear regression equation using age, the NLR, CRP, IL-1β, IL-6, and TNF-α as variables. The results showed the significantly different effects of age, NLR, and IL-6 variations on the plaque load (*p* < 0.05). Further, variations in age and the CRP, NLR, and IL-6 levels also significantly affected the proportion of fibro-lipid tissue in VH-IVUS (*p* < 0.05). Moreover, there was a statistically significantly different effect of variations in TNF-α levels on the area of the necrotic core tissue in VH-IVUS (*p* < 0.05).

**Table 8:** Multifactorial linear regression analysis of plaque load in IVUS.

Characteristics	Beta Coefficient	Standard Error	95% Confidence Interval	*p*
NLR	2.457	0.948	0.138~4.775	0.041 *
Age	−0.425	0.108	−0.689~−0.162	0.008 *
IL-6	−0.664	0.182	−1.109~−0.218	0.011 *

* Statistically significant association between data and outcome.

**Table 9:** Multifactorial linear regression analysis of the proportion of VH-IVUS fibro-lipid tissue.

Characteristics	Beta Coefficient	Standard Error	95% Confidence Interval	*p*
Age	−0.657	0.168	−1.088~0.226	0.011 *
CRP	3.102	0.941	0.683–5.520	0.022 *
IL-6	−0.689	0.271	−1.387~0.009	0.009 *
NLR	3.815	1.472	0.031–7.600	0.049 *

* Statistically significant association between data and outcome.

**Table 10:** Multifactorial linear regression analysis of VH-IVUS necrotic core tissue area.

Characteristics	Beta Coefficient	Standard Error	95% Confidence Interval	*p*
TNF-α	0.02	0.005	0.006~0.034	0.014 *

* Statistically significant association between data and outcome.

## 4. Discussion

PCI implementation has undergone substantial advancements in recent decades, including advancements in the pharmacological coating of stents. The frequency of target vessel revascularization has decreased. The incidence of ISR-related PCIs is around 10% of all PCIs in the United States [2] and has been associated with a higher risk of severe adverse cardiac events compared to PCI procedures performed on new lesions [19,20,21]. In a large contemporary US 2009–2017 report, approximately 50% of PCIs for ISR were performed >2 years after the original stent implantation, and the incidence was more likely after the first year in those who had DES-ISR than in patients with bare-metal-stent (BMS)-ISR [22]. The lumen loss in DES-ISR occurs earlier, often within 1 year after stenting than with BMS. Moreover, the prevalence of newly formed atherosclerosis is more significant when there is an accumulation of lipid-foam-rich macrophages within the neointima, regardless of the presence of necrotic core development and calcification.

The development of ISR involves various components, including anatomical variables such as the blood vessel size, stent factors such as inadequate stent expansion, and clinical factors such as comorbidities and obesity. Several studies have indicated that with the implantation of DES, there is an increased sensitivity to polymers and medicines, inflammation of the blood vessels at the site, and a slower healing process of the injured vessel wall. These factors can contribute to the accelerated aggregation of monocytes and the production of macrophage foam [23]. This phenomenon may also contribute to the development of ISR-induced neo-atherosclerosis.

NLR is an inflammatory marker reflective of the proportional balance between neutrophils and lymphocytes. Neutrophils are involved in the inflammatory response following endothelial injury and in the aggregation of platelet adhesion. In contrast, a low lymphocyte count is associated with poor prognosis among patients with CHD [24,25,26,27,28]. Prior research has directly or indirectly shown the prognostic significance of the NLR for ISR. The NLR is a more reliable measure of the overall inflammatory condition in the body and is less affected by the patient’s physiological condition and CRP levels.

Some scholars now believe that aging is characterized by chronic inflammatory manifestations in the whole body [29,30]. They have discovered that the aging of the vasculature and the senescence of endothelial cells cause intimal dysfunction, early inflammatory activation, and an increased risk of ISR [31,32]. Seo DJ found that the patients from the ISR group were significantly older and had higher hs-CRP levels than those from the non-ISR group [33]. In the present study, we similarly found a higher mean age for patients from the H-NLR group, suggesting that the NLR levels correlate with age and that the increasing age may lead to the active expression of inflammatory factors in vivo, accelerating certain pathologic processes. Myocardial injuries exert stereotyped inflammatory responses. For example, these occurrences activate neutrophils and inflammatory vesicles to produce pro-inflammatory mediators and induce the production of interleukin family members [34,35]. These processes are involved in the resorption of necrotic tissue, the proliferation of fibrous tissue, and the remodeling of the extracellular matrix; these result in structural and functional alterations in the heart. Specialized pro-resolving mediators (SPMs) and interleukins, which are inflammation-related factors, underwent a dynamic evolution in patients with acute myocardial infarction in response to variations in cTnI [36]. The CK-MB, BNP also influences ISR, the NT-proBNP mean corpuscular volume, PLR, TC, LDL-C, and other factors [37,38,39,40,41]. Significant variations in CK-MB, CTnI, BNP, NT-proBNP, and mitral orifice flow velocity A were identified between the L-NLR and H-NLR groups. These results indicate that in vivo myocardial injury and remodeling are influenced by both the elevated NLR and active inflammation.

The Gensini, SYNTAX, and SYNTAX II scores are essential for clinical decision making about precise reperfusion therapy regimens based on coronary anatomy and clinical factors. They are strongly associated with adverse cardiovascular events [42,43]. The SYNTAX II score was reported as an independent risk factor for ISR [44]. A follow-up study of 241 patients undergoing VH-IVUS-guided PCI revealed no significant differences in the pre-PCI plaque composition between the ISR and non-ISR groups. This study discovered that the SYNTAX II score differed substantially between the L-NLR and H-NLR groups, but not the Gensini or SYNTAX scores. Hence, we speculated that in addition to primary coronary artery lesions, NLRs are involved in certain complex physiologic processes that change the creatinine levels and other factors, ultimately causing differences in the SYNTAX II score. Mehran classification is a method to categorize the degree of ISR based on coronary angiographic findings. We found that Mehran staging differed between the two groups, where the L-NLR group was dominated by classes I and II and the H-NLR group by classes II and III.

In contrast to coronary angiography (CAG), an endoluminal imaging technique exemplified by intravascular ultrasonography or optical coherence tomography, the methods mentioned above can yield significant insights into the organization of the vessel wall and the properties of plaques. Endoluminal imaging was suggested by the 2018 ESC/EACTS Guidelines for Myocardial Revascularization to guide the optimal treatment for ISR. Studies have demonstrated that both optical coherence tomography (OCT) and IVUS may improve PCI outcomes in the stent eras [45]. IVUS was superior to soft tissue penetration (5 to 6 mm) compared with OCT (1 to 2 mm), especially in the presence of lipids or a necrotic core. Conversely, one of the most useful features of IVUS that is lacking with OCT is the full-thickness visibility of the vessel wall. By IVUS, it is possible to measure and use vessel size parameters for device sizing, theoretically enabling the achievement of larger stent areas with a low risk of vessel perforation. IVUS-guided PCI was associated with reduced any-type and ischemia-driven target lesion revascularization [46]. However, the published studies are limited to comparisons at the IVUS grayscale level, and it has been shown that the minimum lumen diameter and stent lumen cross-sectional area are independent risk factors for ISR [47].

When compared to paclitaxel (PTX) or sirolimus (SMS), an inhibitor of IL-1β significantly enhanced re-endothelialization and decreased neointimal hyperplasia (NIH) [48]. Prior research has demonstrated that stimulation-induced IL-1β and TNF-α activation can trigger the release of IL-6, which may impact the quantity and functional condition of leucocytes and neutrophils, CRP expression, and blood levels of IL-1β and TNF-α. We observed that the high-NLR group had an upregulated expression of CRP, IL-1β, IL-6, and TNF-α, which confirms that NLR parallels the level of inflammation in vivo. We believe the IL-1β pathway may contribute to the emergence of ISR, although additional validation is necessary in the future.

The IVUS grayscale suggests that the minimum luminal area was smaller and plaque loading was heavier, which indicates that high NLR levels can promote the secretion of inflammatory factors in vivo and mediate the development of neoplastic plaques in the stent. With the development of technologies such as virtual histology, the application of IVUS has further expanded. According to the VH-IVUS analysis, the proportion of fibrous tissue was reduced in the H-NLR group, while that of fibro-lipid tissue was increased. Furthermore, there were variations in the proportions of calcified nodules, the necrotic core, calcified tissue, and VH-TCFA between the two experimental groups. However, there was no statistically significant variation. The proportion of the fiber–lipid component, necrotic core component, calcified nodules, and VH-TCFA are often considered vulnerable plaques [49,50], which are one of the essential causes of acute coronary syndromes. In this study, only the VH-IVUS assay was employed, and there was a deficiency of the measurements of the fibrous cap thickness and liposome volume of restenotic plaques, as well as a lack of investigation into the stenting factors in the mechanism of restenosis. It is hoped that future studies will be conducted to enhance the enrichment and reliability of the conclusions.

Some studies have evaluated how myocardial inflammation post-angioplasty triggers re-infarction during follow-up and post-PCI complications [51]. Inflammation is thought to be a key determinant of plaque instability [52], and the inflammatory response plays an essential role in the development of ISR. A PCI itself is a strong inflammatory stimulant. The immediate systemic reaction to inflammation in a specific area caused by coronary stenting varies significantly across individuals and encourages the development of ISR. Most published research has focused on examining the connection between inflammation and the narrowing of the stent, but has not successfully identified the exact components of the plaque involved. Through employing VH-IVUS, we have identified notable variations in the plaque composition based on inflammation. A heavier plaque load and a higher proportion of vulnerable plaque were associated with high NLR levels. Further, some inflammatory factors were independent risk factors for the plaque load and vulnerable plaque composition. We also underscored the role of the inflammatory response in ISR.

Various factors influence the effectiveness of coronary stents, one of which is restenosis, a severe detrimental disease that compromises the efficacy and prognosis of coronary stents. There are advantages and disadvantages to each drug-coated ball balloon (DCB) and repeat DES implantation. There are still differences between the different types of balloons coated with drugs. We better understood the composition of ISR plaques by using VH-IVUS. As a result, we are able to offer novel perspectives on the fundamental causes of ISR and make informed decisions about therapeutic care. We can now decrease the frequency of adverse cardiovascular events and enhance patient prognosis by selecting various therapeutic options to avoid TLR in ISR.

The study’s limitations include a small sample size, cross-sectional methodology, and a few biases, including the absence of fully occlusive lesions. The deficiency is caused by the lack of a phenotypic assessment of circulating neutrophils in individuals with ISR, primarily centered on measuring the expression of receptors connected to neutrophil adherence to activated platelets and an inflammatory endothelium. Future research is necessary to confirm the inflammatory response concerning the composition of ISR plaques through cellular and in vivo experiments.

## 5. Conclusions

In summary, we observed variations in the composition and stability of the plaque, including the smallest size of the blood vessel, the amount of plaque, the ratio of fibrous tissue to fibro-lipid tissue, the presence of calcified nodules, and the presence of VH-TCFA, between the groups with a low and high NLR. The high NLR group showed a more severe myocardial injury, higher SYNTAX score, higher Mehran staging, heavier plaque load, and higher content of vulnerable components than the low NLR group. In addition, the NLR was a risk factor for the plaque load and the proportion of fibro-lipid tissue in ISR.

## Data Availability

The datasets generated and/or analyzed during the current study are available from the corresponding author upon reasonable request.
